# Transition from zinc salts to trientine tetrahydrochloride in a cohort of adult patients with Wilson disease: the ZICUP study

**DOI:** 10.1186/s13023-026-04311-8

**Published:** 2026-04-03

**Authors:** Aurélia Poujois, Mickael Alexandre Obadia, Nouzha Oussedik-Djebrani, Eduardo Couchonnal-Bedoya, Fabienne Ory-Magne, Dominique Debray

**Affiliations:** 1 Department of Neurology, Rothschild Foundation Hospital, Paris, France; 2 National Reference Center for Wilson Disease and Other Copper-Related Rare Diseases, Rothschild Foundation Hospital, Paris, France; 3 Laboratory of Biological Toxicology, Lariboisière Hospital, Assistance Publique–Hôpitaux de Paris (AP-HP), and Inserm UMR-1144, Université Paris Cité, Paris, France; 4 Department of Paediatric Gastroenterology, Hepatology and Nutrition, Hôpital Femme-Mère-Enfant, Hospices Civils de Lyon, Bron, France; 5 National Reference Center for Wilson Disease and Other Copper-Related Rare Diseases, Femme Mère Enfant Hospital, Hospices Civils de Lyon, Bron, France; 6 Department of Neurology, Purpan Hospital, Toulouse University Hospital (CHU), Toulouse, France

**Keywords:** Wilson disease, Zinc, Trientine, Treatment outcome, Medication adherence, Exchangeable copper

## Abstract

Wilson disease (WD) treatment aims to reduce copper accumulation in the liver and brain. Zinc salts (ZS) are often associated with gastrointestinal side effects, poor adherence, and reduced efficacy. Copper chelators, such as trientine tetrahydrochloride (TETA4), offer an alternative for patients who are intolerant or unresponsive to ZS. This observational study assesses the long-term efficacy and safety of switching from ZS to TETA4 in twenty WD patients (15 hepatic, 5 hepato-neurological) from the French WD Registry. Treatment goals included normalization of aminotransferases, stabilization or improvement of the liver (APRI, liver stiffness and liver ultrasonography; US) and the neurological (Unified Wilson’s Disease Rating Scale; UWDRS) involvement, Clinical Global Impression (CGI) severity scale, and achievement of copper balance targets. Assessments were performed at baseline and every 6 months for 3 years. Adverse events were recorded throughout the follow-up. The results revealed that the main reasons for switching were gastrointestinal intolerance and loss of efficacy with ZS; 60% had elevated ALT at baseline. TETA4 was initiated at a median dose of 412.5 mg (360–450) and titrated up to 750 mg/day (600–900 mg) to achieve the treatment goals. Over 3 years, no changes were observed in ALT, APRI, liver US, UWDRS and CGI scores. TETA4 was well tolerated. In conclusion, transitioning from ZS to TETA4 maintained clinical stability and adherence with a favorable safety profile. Dose adjustments suggest conversion ratios of ~1:6 elemental zinc.

**Supplementary Information:**

The online version contains supplementary material available at 10.1186/s13023-026-04311-8.

## Introduction

Wilson disease (WD) is a rare autosomal recessive genetic disorder that leads to copper overload, primarily in the liver, but also in the eyes and brain. Treatment options, including chelators and zinc salts (ZS), are lifelong and can result in a life expectancy nearly comparable to that of the general population, provided adherence to treatment is maintained [1–3]. Treatment is effective in approximately 80% of patients, with better outcomes observed in cases of hepatic involvement compared to neurological involvement [4–7].

Initial therapy focuses on achieving active copper removal, followed by a maintenance phase during which dosages are typically reduced. ZS are commonly used during the initial phase for asymptomatic patients and neurological patients and during the maintenance phase for stabilized patients [8]. However, poor gastrointestinal tolerance on ZS often leads to poor adherence and reduced efficacy [9–12]. Additionally, mild elevations in serum aminotransferase levels may occur, likely due to impaired copper homeostasis rather than direct hepatotoxicity from zinc [11, 13]. For these reasons, zinc therapy may subsequently need to be replaced by a chelator.

Trientine tetrahydrochloride (TETA4) is a newer formulation with a dual mechanism of action: it promotes urinary excretion of stored copper and inhibits intestinal copper absorption [14]. Available in France since June 2019 under the name of Cuprior®, it is indicated as a second-line therapy for WD patients who are intolerant to D-penicillamine, particularly during the initial phase of the disease, or during the maintenance phase to prevent long-term adverse events associated with D-penicillamine. This chelator has demonstrated efficacy as a maintenance treatment, particularly as a substitute for D-penicillamine [15]. Tolerance is generally good, with only mild digestive and dermatological side effects reported, such as rash and itching [15, 16].

The objective of this study is to assess the efficacy and safety of switching from ZS to TETA4 over a three-year follow-up period in a real-life observational study involving patients enrolled in the national WD registry.

## Patients and methods

### Standard protocol approvals, registrations, and patient consents

This study was conducted by the French National Reference Centre for Wilson Disease in Paris, in collaboration with three university hospitals from the French WD network (Lyon, Besançon, Toulouse). At the time of enrollment in the national WD registry, all patients provided written informed consent for genetic analysis and the anonymous use of their data for research purposes.

WD diagnosis was based on a Leipzig score greater than 4 or the presence of 2 pathogenic mutations in the *ATP7B* gene.

The study protocol was approved by the Institutional Review Board of Hôpitaux Universitaires Paris Nord Val de Seine, Paris 7 University, AP-HP (no. 1343579).

### Study design and patient characteristics

Adults (≥18 years) in the national WD registry treated with ZS (acetate or sulphate) for at least 2 years and switched to TETA4 between October 2019 and January 2021 were included.

According to national guidelines, patients transitioned from maintenance ZS therapy to oral TETA4 (150 mg of trientine base per tablet) over a 2-week period [17]. Prescribing physicians had discretion to determine the initial dose of TETA4, in line with the Summary of Product Characteristics, which recommends a total daily dosage of 450 mg to 975 mg (3 to 6½ film-coated tablets), divided into 2 to 4 doses [18]. Patients were instructed to take TETA4 on an empty stomach, at least 30 minutes before or two hours after meals, and to maintain a minimum one-hour interval between TETA4 and any other medications.

Physicians were advised to follow patients every two months during the first 6 months and every six months thereafter, with dose adjustments based on clinical evaluation, adherence, and laboratory investigations, including serum aminotransferase levels, exchangeable copper (CuEXC), and urinary copper excretion (UCE). Adherence was assessed through physician interviews with patients, clinical examination, liver tests, and copper parameters, and was categorized as high, medium, or low.

Treatment objectives for TETA4 included normalization of serum aminotransferases (AST and ALT < 40 IU/L), stabilization or improvement of neurological symptoms defined as at least a 20% decrease in the Unified Wilson’s Disease Rating Scale (UWDRS) [19], stabilization or improvement on the Clinical Global Impression (CGI) severity scale [20], UCE within the target range of 3–8 µmol/24-hour (150–500 µg/24-hour), and CuEXC within the normal range of 0.65 to 1.1 µmol/L (41–70 µg/L) [21].

### Data collection

Data were collected retrospectively for the 6-month period prior to the switch from ZS to TETA4, and prospectively at baseline (time of switch) and follow-up visits (2, 4, 6, 12, 18, 24, 30, and 36 months).

Variables collected at baseline included demographics, disease duration, and clinical phenotype. At baseline and each visit, assessments included CGI severity score, physician-reported adherence, laboratory parameters (serum aminotransferases, complete blood count, prothrombin time, APRI score, UCE, CuEXC, calculated non-ceruloplasmin-bound copper (NCC)), and TETA4 doses. Neurological status was assessed by a neurologist using the UWDRS, and liver stiffness was evaluated using transient elastography (FibroScan®; Echosens) at baseline and every six months. All adverse events were systematically recorded as part of safety monitoring.

### Data analysis

Quantitative variables were reported as number of patients, median and inter-quartile range (IQR), while categorical variables were expressed as frequencies and percentages. Missing data were not imputed; therefore, the number of patients included in each analysis varied depending on the variable.

Available paired data from more than 10 patients in the cohort at the 3-year follow-up were compared with baseline values using the Wilcoxon signed-rank test. Statistical analyses were performed using R statistical software, and statistical significance was defined as a two-tailed *p* value < 0.05.

For further analyses, patients were divided into two groups based on baseline alanine aminotransferase (ALT) levels:

Group 1, patients (*n* = 7) with normal ALT levels (≤40 IU/L) at baseline.

Group 2, patients (*n* = 13) with elevated ALT levels (>40 IU/l) at baseline.

However, no statistical comparisons between these subgroups were performed because of the small sample size.

## Results

### Main characteristics features of the study population

Main characteristics of the study population are shown in Supplementary Table 1. Twenty patients (6 males) were included at a median age of 41 years (32–48), with a median body mass index (BMI) of 22 kg/m^2^ (20–27), and a median disease duration of 19 years (13–21). Fifteen had a hepatic (H) phenotype, and 5 had a hepato-neurological (HN) phenotype.

Patients received ZS for a mean duration of 11.2 ± 7 years: 16 were treated with zinc acetate (Wilzin® capsules providing 25 or 50 mg elemental zinc) and 4 with zinc sulphate (200-mg capsules containing 23% elemental zinc). The median dose of elemental zinc was 138 mg/day (87.8–150) at transition (Supplementary Table 2).

TETA4 was prescribed as second-line therapy in 6 patients (30%), third-line therapy in 11 patients (55%), and fourth- or fifth-line therapy in 3 patients (15%). The primary reasons for switching from ZS to TETA4 were digestive side effects (e.g., stomach/abdominal pain, nausea, and vomiting) reported by 13 patients (65%), 7 of whom also reported low adherence; and persistently elevated serum aminotransferase levels above 1.5 the upper limit of normal despite high reported adherence, observed in 6 patients (30%). One patient was switched to TETA4 for practical reasons, difficulty accessing zinc acetate, which is only available through hospital pharmacies in France.

### Clinical and biochemical data at baseline

At baseline, AST was elevated (>40 IU/L) in 8/20 patients (40%) (median 52 IU/L, 47–60) and ALT in 13/20 (65%) (median 72 IU/L, 53–94), particularly in those with a hepatic phenotype (Supplementary Table 1). These abnormalities had been present for at least 6 months and remained stable at baseline. Increased liver echogenicity suggestive of steatosis was observed in 12 patients (60%). APRI, liver stiffness measurement (LSM), CGI severity score, and UWDRS score (HN group) were unchanged from values recorded six months earlier.

Patients of group 2 (elevated baseline ALT) had higher CGI severity scores, UCE, and APRI scores while on ZS than patients of group 1 (normal baseline ALT) (Supplementary Table 1). ZS adherence was high in 11 patients (55%), medium in 3, and low in 6.

TETA4 was initiated at a median dose of 412.5 mg/day (320–450 mg) in 2 or 3 divided doses, corresponding to an elemental zinc to TETA4 conversion ratio of 1:3.6 (1:2.9–1:4.2), based on total daily dose (Supplementary Table 2). The TETA4 starting dose was higher in group 2 (Supplementary Table 1). In 10 patients on low-dose elemental zinc (median 83.5 mg (56.3–100)), TETA4 was initiated below the recommended 450 mg/day (median 337.5 mg [300–375]).

### Outcome following transition to TETA4

#### Efficacy

CGI severity and UWDRS (available for 4 out of 5 patients) remained stable between baseline and follow-up assessments over a period of up to three years. No significant changes were observed in serum aminotransferase levels (AST and ALT), median CuEXC values, or APRI scores in the overall cohort (Table 1). As expected, 24 hour-UCE increased after transitioning to TETA4 (Table 1).

**Table 1:** Outcomes up to 3 years following transition from ZS to TETA4

Follow-up time points	Baseline	1 year	2 years	3 years	P*
Number of patients	20	19**	16***	16	
- with normal ALT	7	7	8	7	
- With increased ALT	13	8^b^	8	9	
CGI severity	3 (3–3)	3 (3–3)	3 (2–3.25)	3 (3–3)	ns
BMI (kg/m^2^)	22 (20–27)	22.1 (21.3–27.7)	23.2 (20.7–27.4)^g^	22.8 (21.2–27.1) ^g^	ns
AST (UI/L)	36 (31.5–51)	38 (26.7–60.5)	41.5 (31.7–52.5)^e^	39.5 (26.7–52)^e^	ns
ALT (UI/L)	49 (30.2–73)	47 (32–95)	41 (35.5–87.7)^e^	55.5 (34.5–81.7)^e^	ns
APRI score	0.4 (0.3–0.58)	0.5 (0.35–0.55)^e^	0.45 (0.36–0.68)	0.4 (0.17–0.83)^e^	ns
LSM at TE (kPa)	5.1 (4.4–7.3)^f^	7.4 (6.4–8.6)^c^	7.1 (5–7.7)^c^	7.9 (7–10)^g^	ND
Increased echogenicity at US	12 (60%)	8^c^ (72%)	5^d^ (50%)	8^c^ (100%)	ND
UCE (µmol/24 h)	1.2 (1–1.15)^a^	4.7 (2.8–7.4)^d^	3.7 (2.5–5.8)^a^	4.9 (3.8–7.1)^g^	0.0156
CuEXC (µmol/L)	0.8 (0.5–1)	0.9 (0.8–1)^e^	0.8 (0.6–1)	0.7 (0.6–0.9)	ns
NCC (µmol/L)	0.1 (−0.4–0.7)	−0.4 (−0.6–0.1)^f^	−0.1 (−0.7–0.7)^a^	0.2 (0–0.3)^e^	ns
TETA4 dose (mg/day)	Starting dose 412.5 (320–450)	525 (450–600)	712.5 (487.5–750)	750 (600–900)	0.0003
Adherence to treatment	ZS	TETA4	TETA4	TETA4	ns
- High	11(55%)	14	15	11(69%)	
- Medium	3	3	1	3	
- Low	6	2	2	2	
Adverse events	-	2	0	0	

Group 1 includes patients with normal ALT levels (≤40 U/L) at baseline. Group 2 includes patients with elevated ALT levels (>40 U/l) at baseline

Values are expressed in median (IQR1–IQR3); Missing data: ^a^ *n* = 3; ^b^ *n* = 1; ^c^ *n* = 8; ^d^ *n* = 6; ^e^ *n* = 2; ^f^ *n* = 4; ^g^ *n* = 5

*P values compare measurements at 3 years with baseline when paired data from more than 10 patients were available; *p* < 0.05 was considered statistically significant

**1 patient underwent LT for acute liver failure at 4 months follow-up; *** 1 patient was switched to D-penicillamine within 1- and 2-years follow-up

Abbreviations: CGI, clinical global impression scale; BMI, body mass index; AST, aspartate aminotransferase; ALT, alanine aminotransferase; APRI, AST-to platelet ratio index; LSM, liver stiffness measurement; TE, transient elastography; UCE, urinary copper excretion; CuEXC, exchangeable copper; NCC, non-ceruloplasmin bound copper; ZS, zinc salts; TETA4, Trientine 4-HCL; ND, not done

**Fig. 1:**
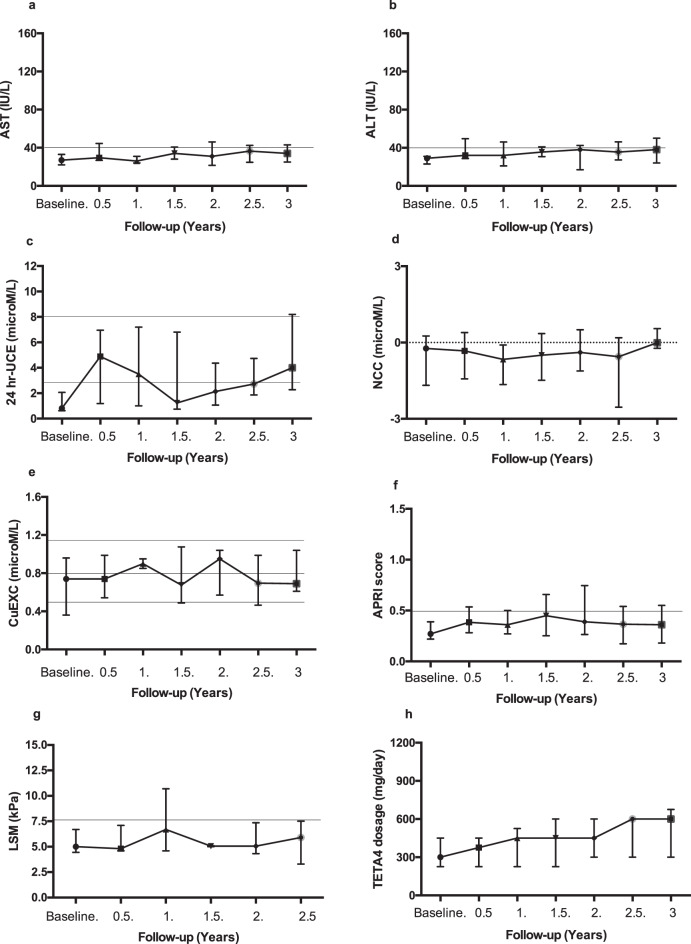
Group 1 – Patients with normal ALT at baseline: evolution of biological markers and TETA4 doses

**Fig. 2:**
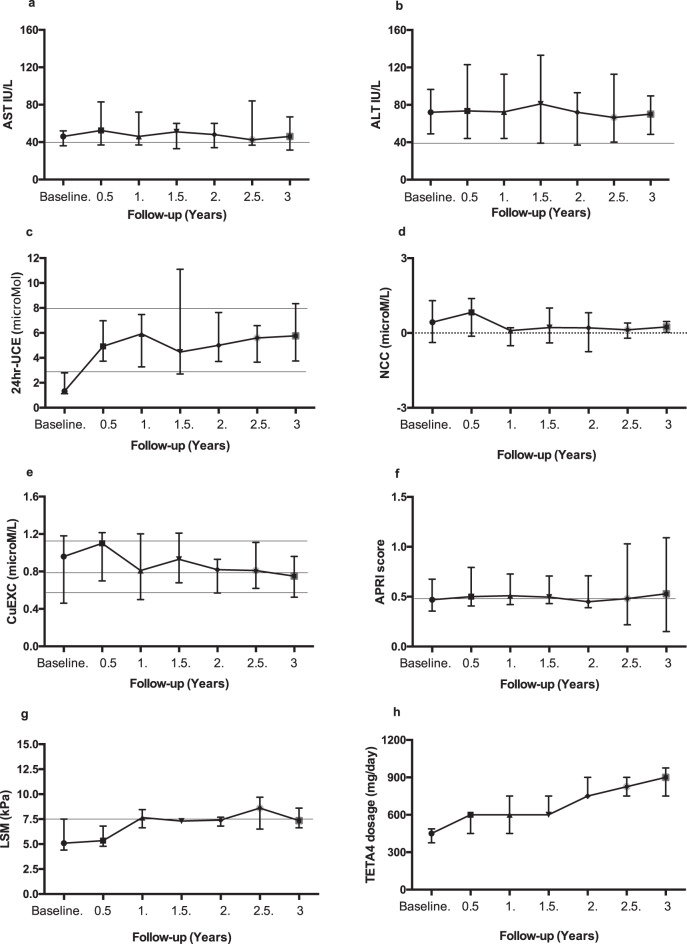
Group 2 – Patients with elevated ALT at baseline: evolution of biological markers and TETA4 doses

LSM appeared to increase during the first year following initiation of TETA4 and subsequently stabilized. Statistical analysis was not performed due to the limited availability of paired data. Figures 1 and 2 illustrate the evolution of these parameters in group 1 and group 2 respectively between baseline and subsequent evaluations up to the 3-year follow-up.

#### Dose adjustments

In an effort to optimize copper balance and maintain the aminotransferase levels in the normal range, the TETA4 dose was progressively increased over time (Table 1). By the three-year follow-up, a median dose of 750 mg/day was reached, which was associated with maintenance of CuEXC levels at a median level of 0.7 μM/l (Table 1). The required median dose was higher in patients of group 2, reaching 900 mg/day (Fig. 2h). The calculated conversion ratio from elemental zinc to TETA4 based on total daily dose was 1:6 mg (1:4.7–1:7.1) at 3 years (Supplementary Table 2).

Adherence to treatment did not mprove over time (Table 1).

#### Safety

Overall, TETA4 was well tolerated. Two patients reported digestive issues: one experienced constipation, and another developed a digestive abscess due to diverticulitis (at 6 months).

Antinuclear antibodies (ANA) were present at baseline in 4 patients. During the first 2 years of follow-up, 3 additional patients developed newly positive ANA. At the 3-year follow-up, ANA remained present in 4 patients. No cases of autoimmune disorders were reported.

Among the 20 patients, 2 had unfavorable outcomes. One patient with severe depression, was nonadherent to both ZS and TETA4, progressed to liver failure and required liver transplantation after 4 months. The other patient, with a HN phenotype, showed neurological deterioration with a slight increase in UWDRS and CuEXC, after 2 years of TETA4 at 750 mg/day. The patient was subsequently switched to D-Penicillamine but continued to experience neurological fluctuations, likely related to poor treatment adherence.

## Discussion

This is the first real-world study to examine the natural history of patients with WD who transitioned from ZS to TETA4 during maintenance therapy, with monitoring for up to 3 years. TETA4 was well tolerated; serious adverse events occurred only in the context of non-adherence, a key issue in patients switching from ZS due to gastrointestinal intolerance. These findings are consistent with the established safety profile of TETA4 as maintenance therapy [15, 16].

Efficacy was reflected by overall clinical stability, with no longitudinal changes in AST, ALT, or APRI. Treatment adherence, a major challenge in WD with non-adherence rates reaching up to 30% [22], did not appear to improve sustainably after switching from ZS to TETA4.

At transition, up to 60% of patients on ZS had elevated aminotransferase levels (1.5 to 2 times the upper limit of normal). Mild serum aminotransferase elevations during ZS therapy are commonly linked to suboptimal control of copper balance and poor adherence [9, 23, 24]. Of the 9 patients (45%) with medium or low adherence to ZS, 6 had elevated aminotransferase levels at baseline. Higher baseline UCE levels (i.e., while on ZS) in patients with elevated ALT (group 2) than in those with normal ALT (group 1) suggest suboptimal adherence and possibly an increased hepatic copper burden.

Despite clinical stability, switching to TETA4 did not normalize or improve aminotransferases over 3 years. BMI remained normal throughout; alcohol intake was not assessed. Although 24-hour UCE values generally fell within recommended targets, robust evidence validating these targets for trientine is limited, and optimal CuEXC targets for maintenance therapy have yet to be established [8]. Preliminary data from our center suggest that CuEXC 0.4–0.8 µmol/L may be associated with remission. Following a median 100% increase in the TETA4 dose, CuEXC values generally fell below 0.8 µmol/L by year 3 at a median dose of 750 mg/day, suggesting that the initial dosing may have been suboptimal.

No formal recommendation exists for switching from ZS to TETA4. The low starting doses used here were based on prior experience with trientine 2HCL and on the 1:0.64 conversion ratio from trientine 2HCL to TETA4 proposed in the Triumph study [25].

Based on 3-year follow-up doses, our data suggest a potential 1:6 elemental zinc toTETA4 conversion ratio on a daily dose basis, which requires confirmation. The sample size is too small to support tailored recommendations according to baseline aminotransferase levels (normal vs. elevated). Nevertheless, the median TETA4 requirement of 900 mg/day in patients with elevated ALT aligns with the 1:1 D-penicillamine to TETA4 conversion ratio reported in the Chelate study [1, 5].

A key limitation is the small sample size, inherent to rare diseases such as WD, which has a prevalence of approximately 1 in 30,000 individuals [26]. Additionally, this was an observational study with flexible dosing. Nevertheless, follow-up was standardized through the French WD network and national registry.

In conclusion, this real-world study suggests that switching from ZS to TETA4 for gastrointestinal intolerance improves tolerability and maintains clinical stability, although liver tests may not improve in patients with elevated baseline transaminases. This may reflect initial underdosing of TETA4. A provisional zinc to TETA4 conversion ratio of approximately 1:6 is proposed. These findings underscore the need for individualized dosing and close monitoring and may help inform future guidelines for transitioning patients with WD from ZS to trientine. Prospective randomized studies are needed to confirm these observations.

## Electronic supplementary material

Below is the link to the electronic supplementary material.

Supplementary Material 1

Supplementary Material 2

## Data Availability

The datasets used and/or analyzed during the current study are available from the corresponding author on reasonable request
