# Supplementary material for: Transition from zinc salts to trientine tetrahydrochloride in a cohort of adult patients with Wilson disease: the ZICUP study
Source: Orphanet J Rare Dis. 2026 Apr 3;21:200. doi: 10.1186/s13023-026-04311-8 (PMC13173772; doi:10.1186/s13023-026-04311-8)
Supplement: Supplementary file 1 — Supplementary Material 1 [file 13023_2026_4311_MOESM1_ESM.docx]

**Supplementary Table 1:** Cohort characteristics before conversion from zinc salts to trientine tetrahydrochloride (TETA4)

|  | Six months before baseline | At baseline | | |
| --- | --- | --- | --- | --- |
|  | Study cohort | Study cohort | Group 1* | Group 2** |
| Number of patients | 20 | 20 | 7 | 13 |
| Age (years) | 41 (32-48) | 41 (32-48) | 40 (37.2-46.9) | 42 (28.2-47.3) |
| Clinical phenotype | 15H /5HN | 15H /5HN | 4H /3HN | 11H /2HN |
| Disease duration (years) | 19 (13-21) | 19(13-21) | 19 (14-20.5) | 19 (13-21) |
| Ongoing treatment  *- Zinc acetate (n patients)*  *- Zinc sulphate (n patients)*  *Duration of ZS therapy (years)*  Doses of elemental Zinc  (mg/day) | 16  4  10.5 (4.4-16.4)  138 (87.8-150) | 16  4  11 (5-17)  138 (87.8-150) | 7  0  11 (6.4-15)  100 (50-150) | 9  4  6.5 (5-17)  150 (100-150) |
| Adherence to ZS (N patients by clinical phenotype)  - High  - Medium  - Low | 11 (8H; 3HN)  5 (4H; 1HN)  4 (3H; 1HN) | 11 (8H; 3HN)  3 (2H; 1HN)  6 (5H; 1HN) | 4 (3H; 1HN)  1 (1HN)  2 (1H; 1HN) | 7 (5H; 2HN)  2 (2H)  4 (4H) |
| Reasons for conversion to TETA4  Gastric intolerance  Increased liver enzymes  Practical issue | -  -  - | 13  6  1 | 4  2  1 | 9  4  0 |
| BMI (kg/m^2^) | 22 (20-27) | 22(20-27) | 21.6 (20.6-25.2) | 24.9 (20.1-27) |
| UWDRS *** | 25 (10-37) | 23 (10-37) | 41 (25.5-50.5) | 30 (26.5-33.5) |
| CGI severity | 3 (2.8-3) | 3 (3-3) | 2 (2-3) | 3 (3-3) |
| UCE (µmol/L) | 1 (0.5-1.8) ^a^ | 0.9 (0.7-1.4) ^f^ | 0.6 (0.5-0.8) ^i^ | 1.1 (0.8-1.5) ^h^ |
| UCE (µmol/24h) | 1.2 (0.8-2.2) ^b^ | 1.2 (1-1.15) ^h^ | 0.8 (0.7-1) ^h^ | 1.3 (1.2-2.2) ^j^ |
| CuEXC (µmol/L) | 0.7 (0.6-1.) ^c^ | 0.8 (0.5-1) | 0.7 (0.5-0.9) | 1 (0.6-1.2) |
| NCC (µmol/L) | 0.2 (-0.3-0.4) ^d^ | 0.1 (-0.4-0.7) ^d^ | -0.2 (-0.9-0.1) ^i^ | 0.4 (-0.1-1.1) ^h^ |
| AST (UI) | 36.5 (31.3-49) | 36 (31.5-51) | 27 (22.5-32) | 46 (38-52) |
| ALT (UI) | 47 (33.8-83.5) | 49 (30.2-73) | 29 (24.5-30.5) | 72 (53-94) |
| APRI score | 0.4 (0.3-0.6) | 0.4 (0.3-0.58) | 0.3 (0.2-0.4) | 0.5 (0.4-0.6) |
| LSM by Transient elastography (kPa) | 5.9 (4.8-7.2) ^e^ | 5.1 (4.4-7.3) ^a^ | 5 (4.9-6) ^c^ | 5.1 (4.4-7.4) ^c^ |
| Increased echogenicity at US | 12 (60%) | 12 (60%) | 4 (57%) | 8 (61.5%) |
| Starting dose of TETA4 (mg/day) | - | 412.5 (320-450) | 300 (262.5-412.5) | 450 (375-450) |

*Group 1 includes patients with normal ALT levels (≤40 U/L) at baseline; **Group 2 includes patients with elevated ALT levels (> 40U/l) at baseline.

Values are expressed in median (IQR1-IQR3); Missing data: ^a^, n=4; ^b^, n=8; ^c^, n=2; ^d^, n=4; ^e^, n=5; ^f^, n=4; ^g^, n=9; ^h^, n=3; ^i^, n=1^;^ ^j^, n=6.

*** Restricted to patients with the hepato-neurological clinical phenotype

Abbreviations: ZS, Zinc salts; TETA4, Trientine 4-HCL; H, hepatic; HN, hepato-neurological; BMI, body mass index; UWDRS, unified Wilson disease rating scale; CGI, clinical global impression scale; UCE, urinary copper excretion; CuEXC, exchangeable copper; NCC, non-ceruloplasmin bound copper; AST, aspartate aminotransferase; ALT, alanine aminotransferase; APRI, AST-to platelet ratio index; LSM, liver stiffness measurement; US, ultrasonography.
