# Supplementary material for: Transition from zinc salts to trientine tetrahydrochloride in a cohort of adult patients with Wilson disease: the ZICUP study
Source: Orphanet J Rare Dis. 2026 Apr 3;21:200. doi: 10.1186/s13023-026-04311-8 (PMC13173772; doi:10.1186/s13023-026-04311-8)
Supplement: Supplementary file 2 — Supplementary Material 2 [file 13023_2026_4311_MOESM2_ESM.docx]

**Supplementary Table 2**: Trientine 4-HCL doses and conversion ratios from elemental Zinc (baseline to 3-year follow-up)

|  | Baseline | Year 1 | Year 2 | Year 3 |
| --- | --- | --- | --- | --- |
| Number of patients | 20 | 19 | 17 | 16 |
| Elemental Zinc  (mg/day) * | 138 (87.8-150) | - | - | - |
| TETA4 dose  (mg/day) | 412.5 (320-450) | 525 (450-600) | 712.5 (487.5-750) | 750 (600-900) |
| Percent increase in TETA4 dose from baseline (%) | - | 20 (0-58.3) | 66.7 (27.1-100) | 100 (45.8-100) |
| Elemental Zinc to TETA4 conversion ratio | 1:3.6 (2.9-4.2) | 1:4 (3.4-5) | 1: 5.2 (4.6-6) | 1: 6 (4.7-7.1) |

* either Zinc acetate or Zinc Sulphate

Values are expressed in median (IQR1-IQR3)

Abbreviations: TETA4, Trientine-4HCL
